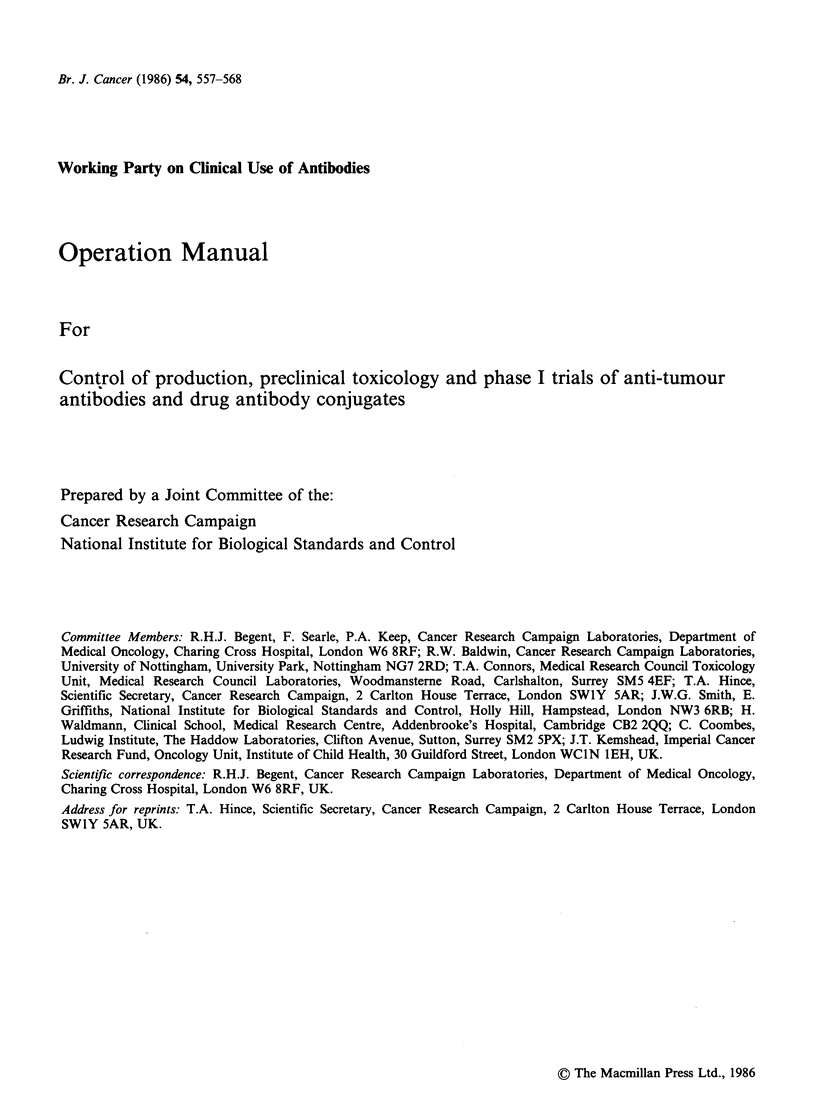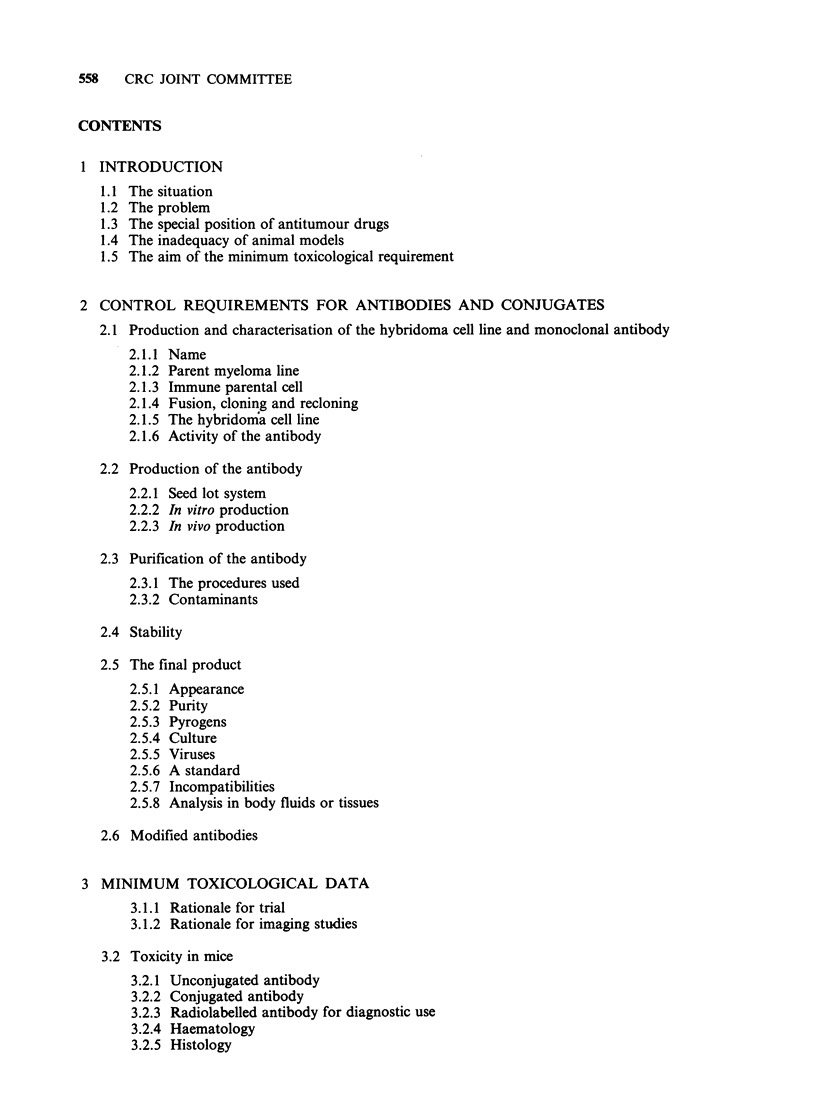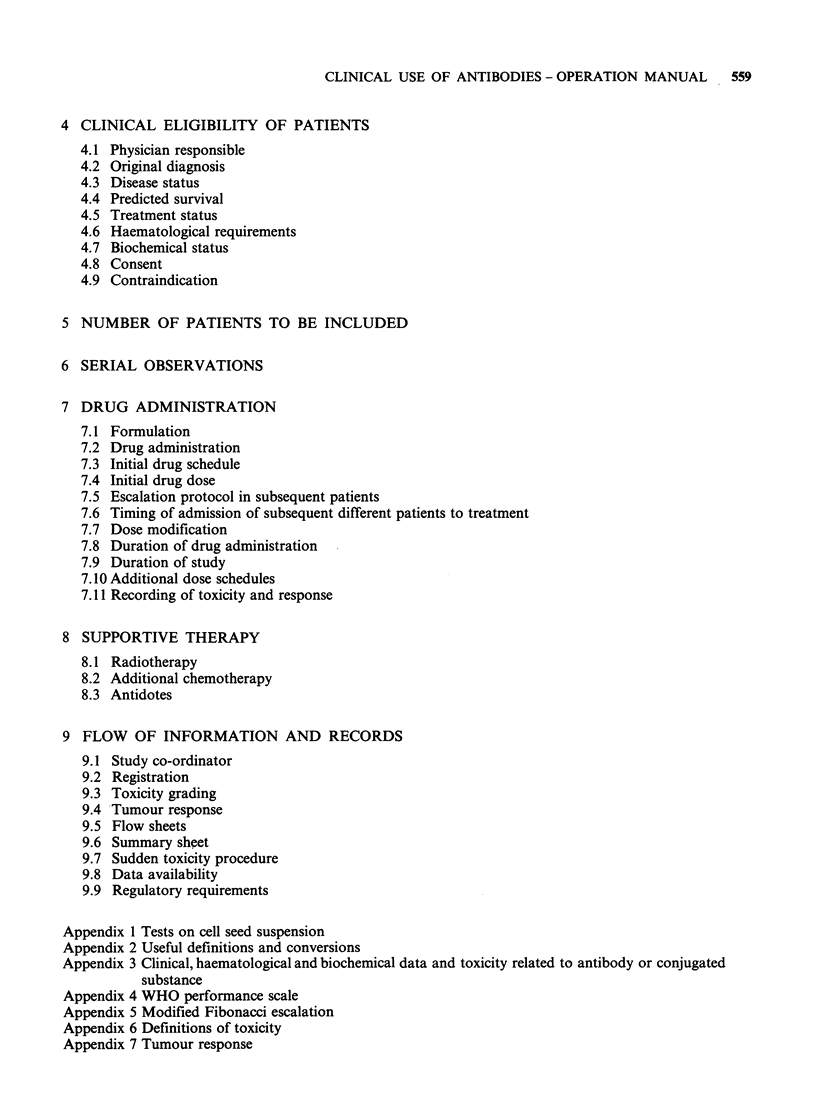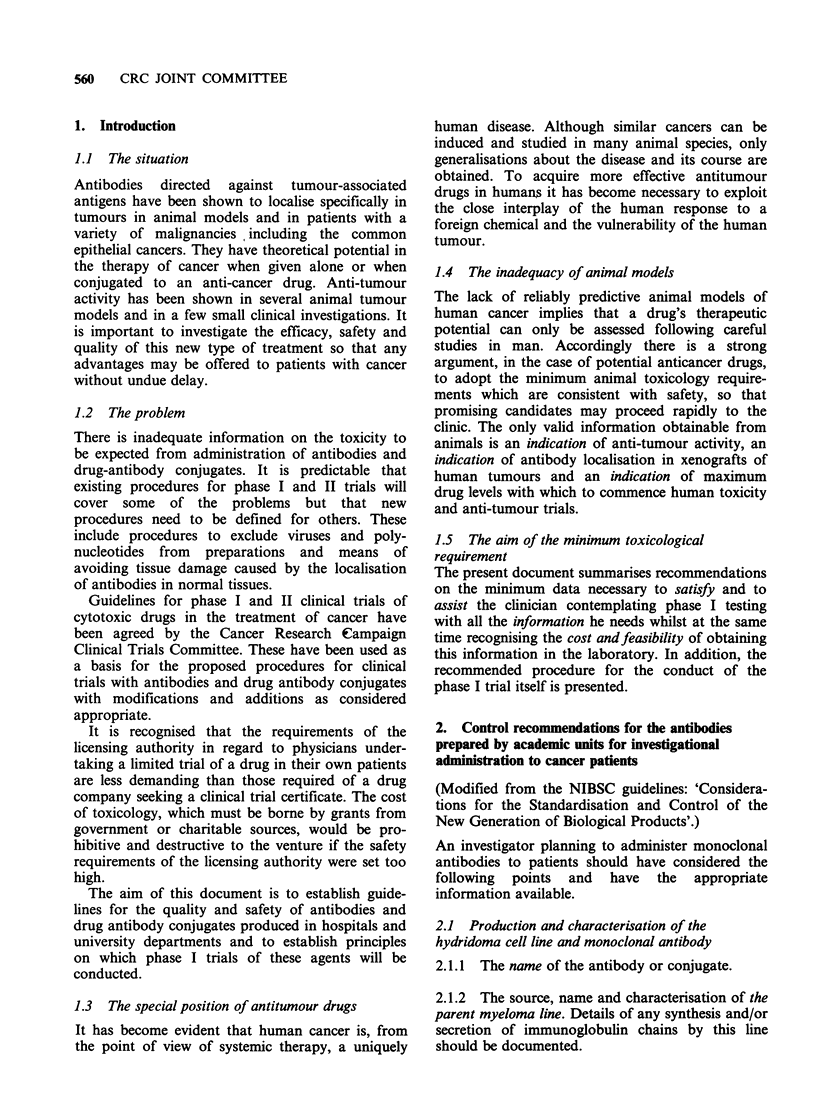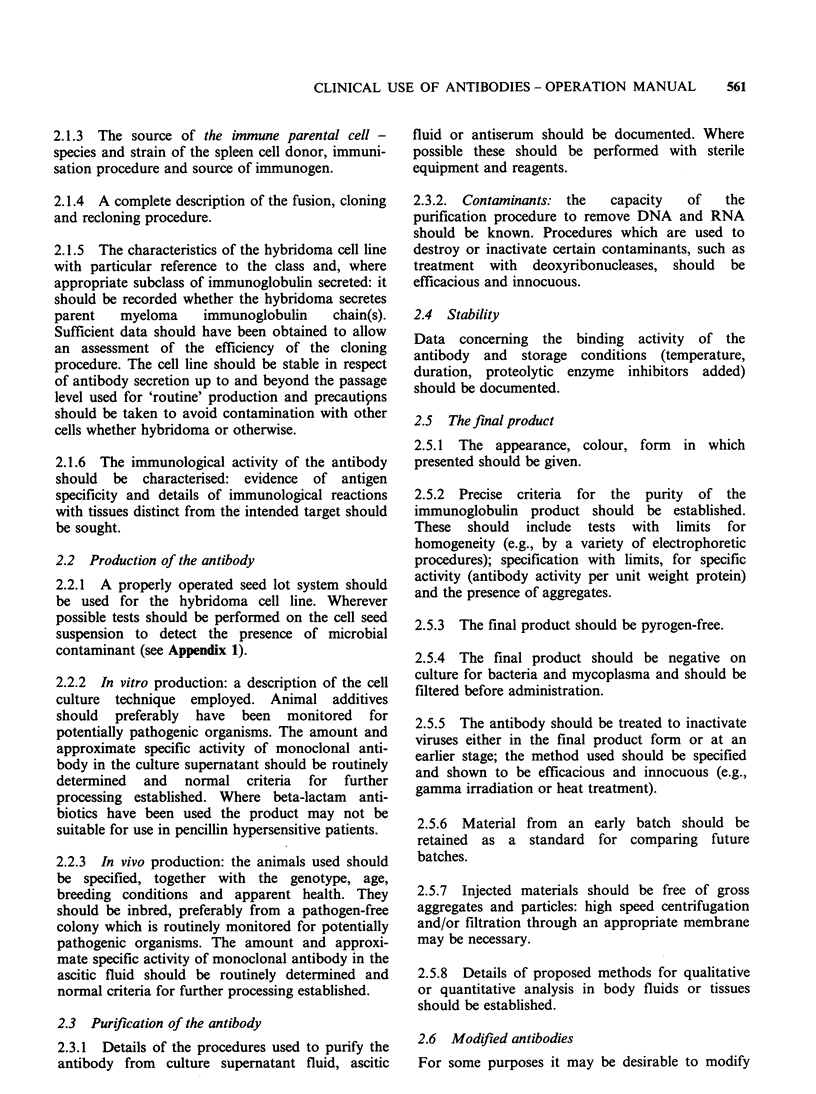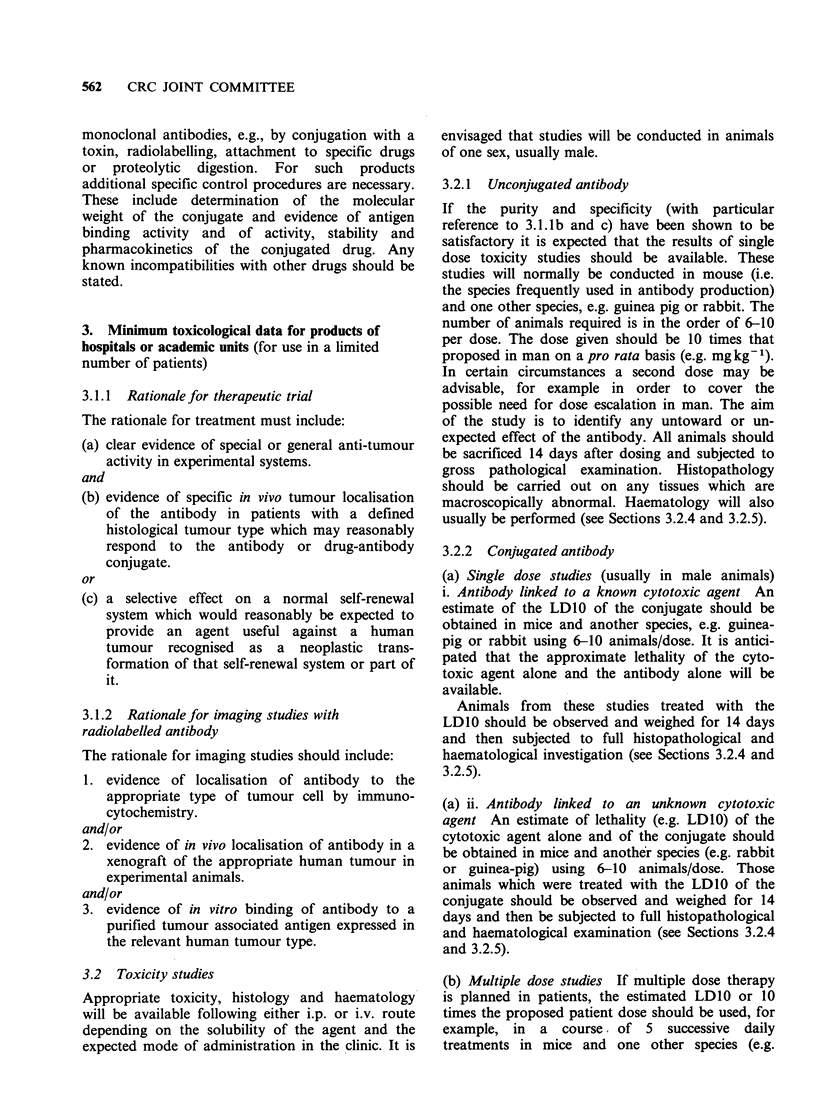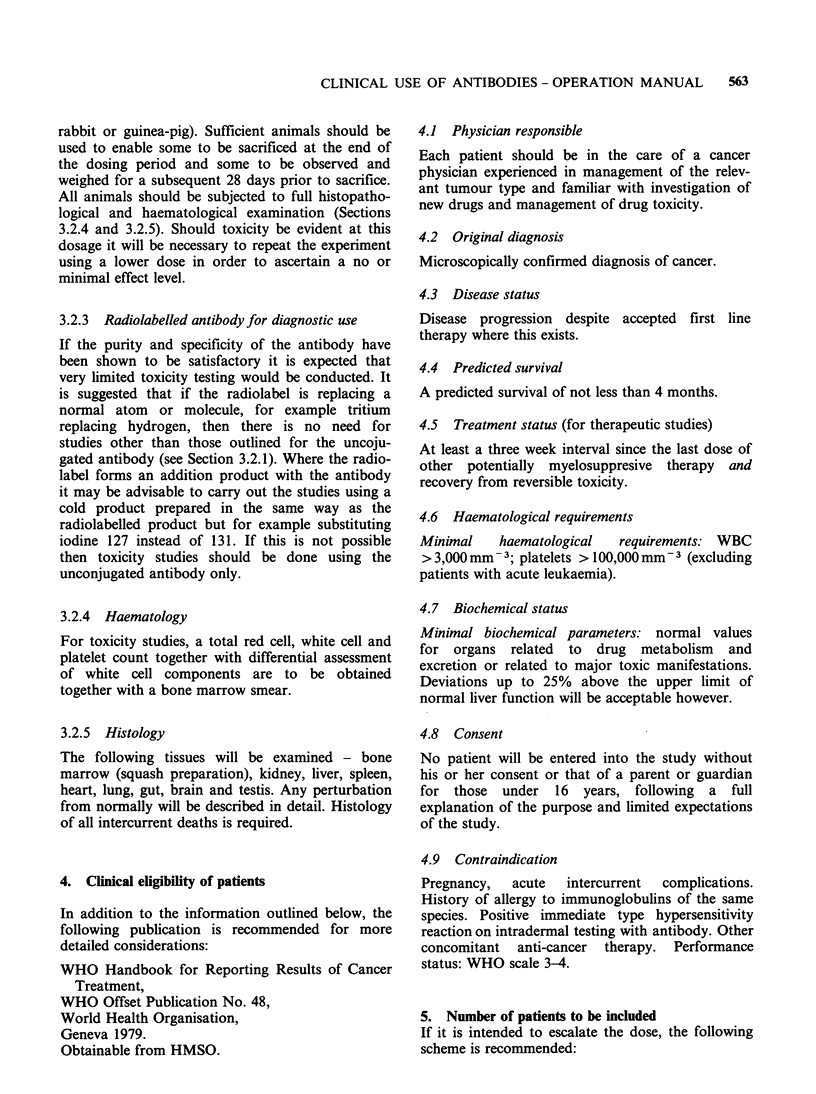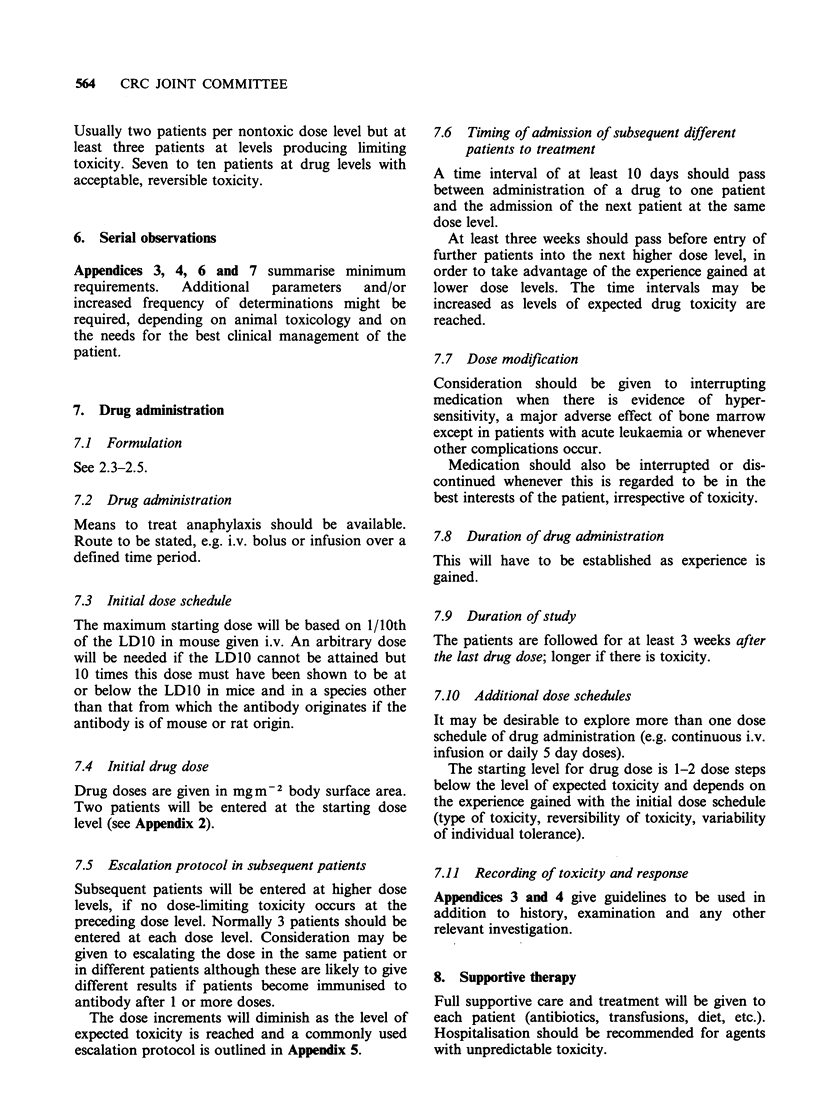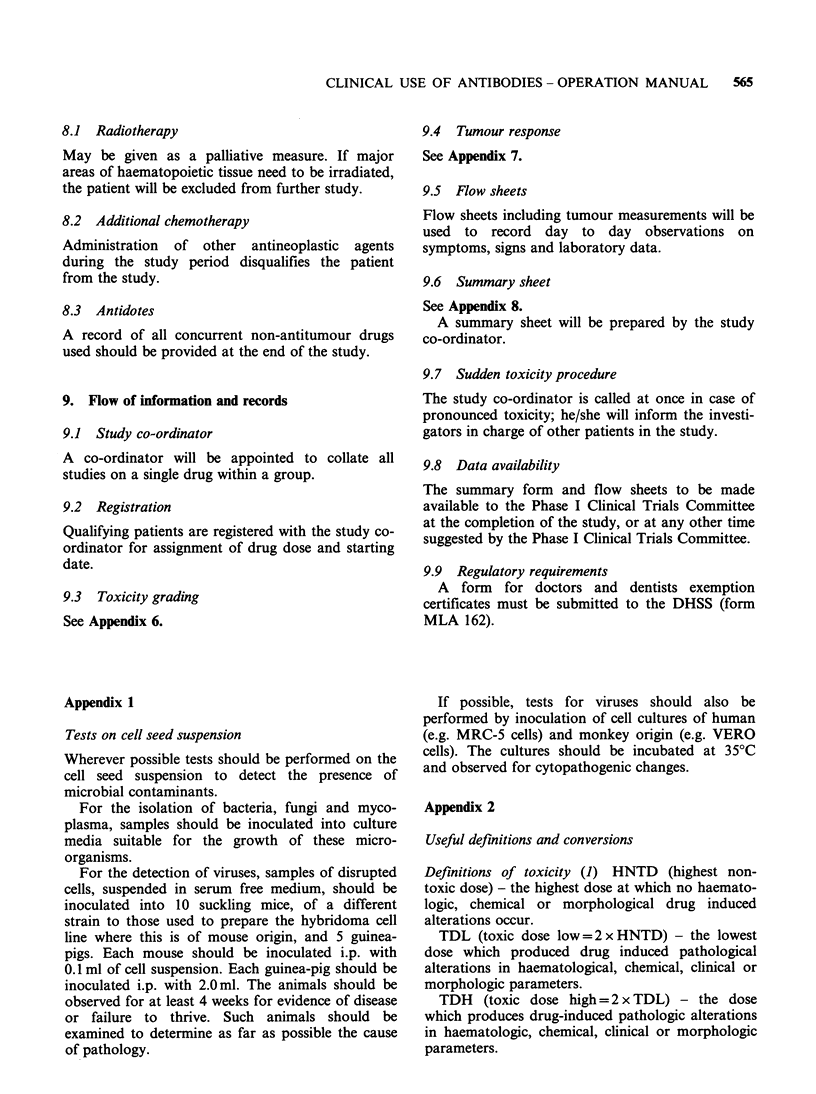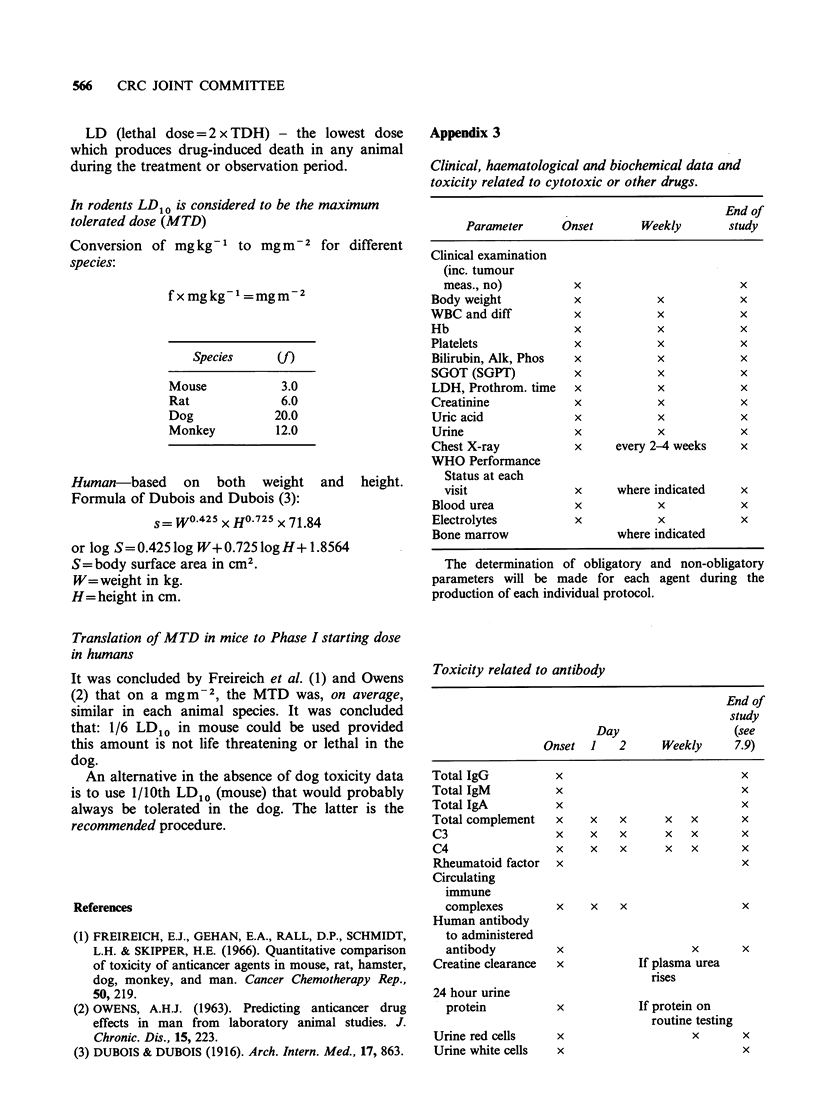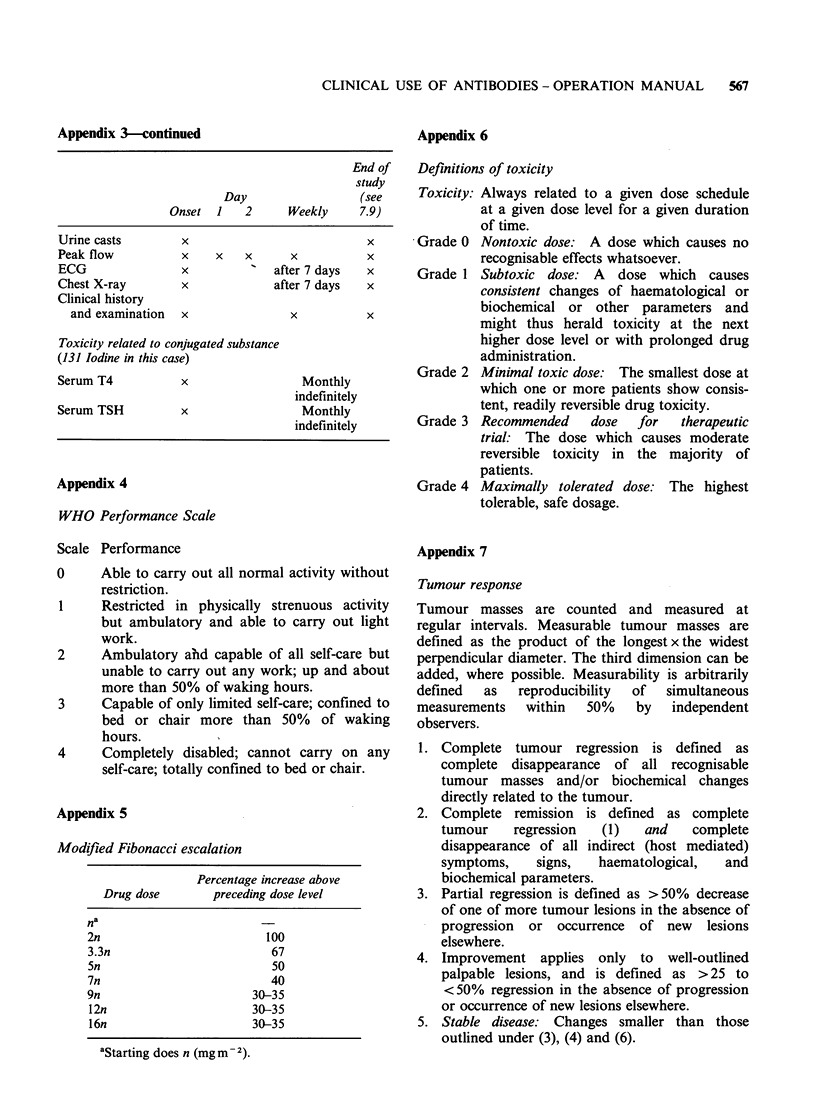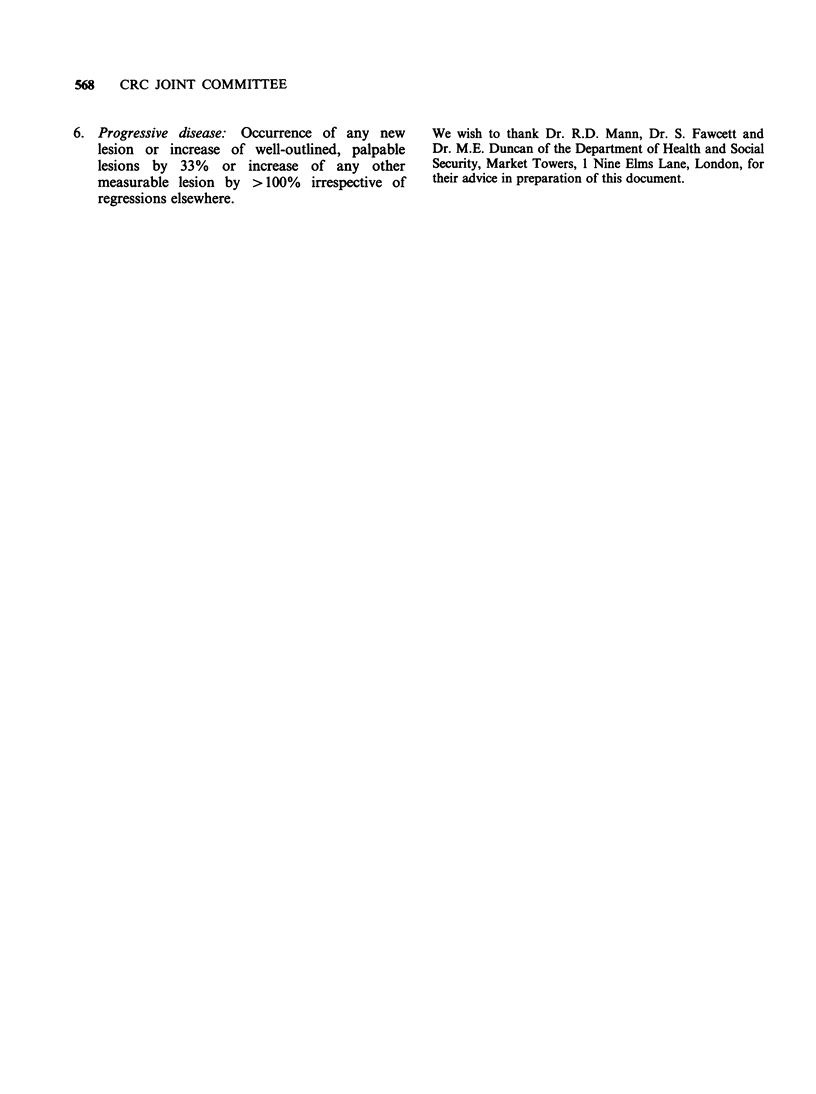# Operation manual for control of production, preclinical toxicology and phase I trials of anti-tumour antibodies and drug antibody conjugates. Prepared by a Joint Committee of the: Cancer Research Campaign National Institute for Biological Standards and Control.

**DOI:** 10.1038/bjc.1986.209

**Published:** 1986-09

**Authors:** 


*Br. J. Cancer* (1986) **54**, 557–568

**Working Party on Clinical Use of Antibodies**

### Operation Manual

For

Control of production, preclinical toxicology and phase I trials of anti-tumour antibodies and drug antibody conjugates

Prepared by a Joint Committee of the:

Cancer Research Campaign

National Institute for Biological Standards and Control

*Committee Members:* R.H.J. Begent, F. Searle, P.A. Keep, Cancer Research Campaign Laboratories, Department of Medical Oncology, Charing Cross Hospital, London W6 8RF; R.W. Baldwin, Cancer Research Campaign Laboratories, University of Nottingham, University Park, Nottingham NG7 2RD; T.A. Connors, Medical Research Council Toxicology Unit, Medical Research Council Laboratories, Woodmansterne Road, Carlshalton, Surrey SM5 4EF; T.A. Hince, Scientific Secretary, Cancer Research Campaign, 2 Carlton House Terrace, London SW1Y 5AR; J.W.G. Smith, E. Griffiths, National Institute for Biological Standards and Control, Holly Hill, Hampstead, London NW3 6RB; H. Waldmann, Clinical School, Medical Research Centre, Addenbrooke's Hospital, Cambridge CB2 2QQ; C. Coombes, Ludwig Institute, The Haddow Laboratories, Clifton Avenue, Sutton, Surrey SM2 5PX; J.T. Kemshead, Imperial Cancer Research Fund, Oncology Unit, Institute of Child Health, 30 Guildford Street, London WC1N 1EH, UK.

*Scientific correspondence:* R.H.J. Begent, Cancer Research Campaign Laboratories, Department of Medical Oncology, Charing Cross Hospital, London W6 8RF, UK.

*Address for reprints:* T.A. Hince, Scientific Secretary, Cancer Research Campaign, 2 Carlton House Terrace, London SW1Y 5AR, UK.

© The Macmillan Press Ltd., 1986

### CONTENTS

1 INTRODUCTION

1.1 The situation
1.2 The problem
1.3 The special position of antitumour drugs
1.4 The inadequacy of animal models
1.5 The aim of the minimum toxicological requirement

2 CONTROL REQUIREMENTS FOR ANTIBODIES AND CONJUGATES

2.1 Production and characterisation of the hybridoma cell line and monoclonal antibody

2.1.1 Name
2.1.2 Parent myeloma line
2.1.3 Immune parental cell
2.1.4 Fusion, cloning and recloning
2.1.5 The hybridoma cell line
2.1.6 Activity of the antibody

2.2 Production of the antibody

2.2.1 Seed lot system
2.2.2 *In vitro* production
2.2.3 *In vivo* production

2.3 Purification of the antibody

2.3.1 The procedures used
2.3.2 Contaminants

2.4 Stability

2.5 The final product

2.5.1 Appearance
2.5.2 Purity
2.5.3 Pyrogens
2.5.4 Culture
2.5.5 Viruses
2.5.6 A standard
2.5.7 Incompatibilities
2.5.8 Analysis in body fluids or tissues

2.6 Modified antibodies

3 MINIMUM TOXICOLOGICAL DATA

3.1.1 Rationale for trial
3.1.2 Rationale for imaging studies

3.2 Toxicity in mice

3.2.1 Unconjugated antibody
3.2.2 Conjugated antibody
3.2.3 Radiolabelled antibody for diagnostic use
3.2.4 Haematology
3.2.5 Histology

4 CLINICAL ELIGIBILITY OF PATIENTS

4.1 Physician responsible
4.2 Original diagnosis
4.3 Disease status
4.4 Predicted survival
4.5 Treatment status
4.6 Haematological requirements
4.7 Biochemical status
4.8 Consent
4.9 Contraindication

5 NUMBER OF PATIENTS TO BE INCLUDED

6 SERIAL OBSERVATIONS

7 DRUG ADMINISTRATION

7.1 Formulation
7.2 Drug administration
7.3 Initial drug schedule
7.4 Initial drug dose
7.5 Escalation protocol in subsequent patients
7.6 Timing of admission of subsequent different patients to treatment
7.7 Dose modification
7.8 Duration of drug administration
7.9 Duration of study
7.10 Additional dose schedules
7.11 Recording of toxicity and response

8 SUPPORTIVE THERAPY

8.1 Radiotherapy
8.2 Additional chemotherapy
8.3 Antidotes

9 FLOW OF INFORMATION AND RECORDS

9.1 Study co-ordinator
9.2 Registration
9.3 Toxicity grading
9.4 Tumour response
9.5 Flow sheets
9.6 Summary sheet
9.7 Sudden toxicity procedure
9.8 Data availability
9.9 Regulatory requirements

Appendix 1 Tests on cell seed suspension
Appendix 2 Useful definitions and conversions
Appendix 3 Clinical, haematological and biochemical data and toxicity related to antibody or conjugated substance
Appendix 4 WHO performance scale
Appendix 5 Modified Fibonacci escalation
Appendix 6 Definitions of toxicity
Appendix 7 Tumour response

### 1. Introduction

### 1.1 The situation

Antibodies directed against tumour-associated antigens have been shown to localise specifically in tumours in animal models and in patients with a variety of malignancies including the common epithelial cancers. They have theoretical potential in the therapy of cancer when given alone or when conjugated to an anti-cancer drug. Anti-tumour activity has been shown in several animal tumour models and in a few small clinical investigations. It is important to investigate the efficacy, safety and quality of this new type of treatment so that any advantages may be offered to patients with cancer without undue delay.

### 1.2 The problem

There is inadequate information on the toxicity to be expected from administration of antibodies and drug-antibody conjugates. It is predictable that existing procedures for phase I and II trials will cover some of the problems but that new procedures need to be defined for others. These include procedures to exclude viruses and polynucleotides from preparations and means of avoiding tissue damage caused by the localisation of antibodies in normal tissues.

Guidelines for phase I and II clinical trials of cytotoxic drugs in the treatment of cancer have been agreed by the Cancer Research Campaign Clinical Trials Committee. These have been used as a basis for the proposed procedures for clinical trials with antibodies and drug antibody conjugates with modifications and additions as considered appropriate.

It is recognised that the requirements of the licensing authority in regard to physicians undertaking a limited trial of a drug in their own patients are less demanding than those required of a drug company seeking a clinical trial certificate. The cost of toxicology, which must be borne by grants from government or charitable sources, would be prohibitive and destructive to the venture if the safety requirements of the licensing authority were set too high.

The aim of this document is to establish guidelines for the quality and safety of antibodies and drug antibody conjugates produced in hospitals and university departments and to establish principles on which phase I trials of these agents will be conducted.

### 1.3 The special position of antitumour drugs

It has become evident that human cancer is, from the point of view of systemic therapy, a uniquely human disease. Although similar cancers can be induced and studied in many animal species, only generalisations about the disease and its course are obtained. To acquire more effective antitumour drugs in humans it has become necessary to exploit the close interplay of the human response to a foreign chemical and the vulnerability of the human tumour.

### 1.4 The inadequacy of animal models

The lack of reliably predictive animal models of human cancer implies that a drug's therapeutic potential can only be assessed following careful studies in man. Accordingly there is a strong argument, in the case of potential anticancer drugs, to adopt the minimum animal toxicology requirements which are consistent with safety, so that promising candidates may proceed rapidly to the clinic. The only valid information obtainable from animals is an *indication* of anti-tumour activity, an *indication* of antibody localisation in xenografts of human tumours and an *indication* of maximum drug levels with which to commence human toxicity and anti-tumour trials.

### 1.5 The aim of the minimum toxicological requirement

The present document summarises recommendations on the minimum data necessary to *satisfy* and to *assist* the clinician contemplating phase I testing with all the *information* he needs whilst at the same time recognising the *cost and feasibility* of obtaining this information in the laboratory. In addition, the recommended procedure for the conduct of the phase I trial itself is presented.

### 2. Control recommendations for the antibodies prepared by academic units for investigational administration to cancer patients

(Modified from the NIBSC guidelines: 'Considerations for the Standardisation and Control of the New Generation of Biological Products'.)

An investigator planning to administer monoclonal antibodies to patients should have considered the following points and have the appropriate information available.

### 2.1 Production and characterisation of the hydridoma cell line and monoclonal antibody

2.1.1 The *name* of the antibody or conjugate.

2.1.2 The source, name and characterisation of *the parent myeloma line*. Details of any synthesis and/or secretion of immunoglobulin chains by this line should be documented.

2.1.3 The source of *the immune parental cell* – species and strain of the spleen cell donor, immunisation procedure and source of immunogen.

2.1.4 A complete description of the fusion, cloning and recloning procedure.

2.1.5 The characteristics of the hybridoma cell line with particular reference to the class and, where appropriate subclass of immunoglobulin secreted: it should be recorded whether the hybridoma secretes parent myeloma immunoglobulin chain(s). Sufficient data should have been obtained to allow an assessment of the efficiency of the cloning procedure. The cell line should be stable in respect of antibody secretion up to and beyond the passage level used for 'routine' production and precautions should be taken to avoid contamination with other cells whether hybridoma or otherwise.

2.1.6 The immunological activity of the antibody should be characterised: evidence of antigen specificity and details of immunological reactions with tissues distinct from the intended target should be sought.

*2.2 Production of the antibody*

2.2.1 A properly operated seed lot system should be used for the hybridoma cell line. Wherever possible tests should be performed on the cell seed suspension to detect the presence of microbial contaminant (see **Appendix 1**).

2.2.2 *In vitro* production: a description of the cell culture technique employed. Animal additives should preferably have been monitored for potentially pathogenic organisms. The amount and approximate specific activity of monoclonal antibody in the culture supernatant should be routinely determined and normal criteria for further processing established. Where beta-lactam antibiotics have been used the product may not be suitable for use in pencillin hypersensitive patients.

2.2.3 *In vivo* production: the animals used should be specified, together with the genotype, age, breeding conditions and apparent health. They should be inbred, preferably from a pathogen-free colony which is routinely monitored for potentially pathogenic organisms. The amount and approximate specific activity of monoclonal antibody in the ascitic fluid should be routinely determined and normal criteria for further processing established.

*2.3 Purification of the antibody*

2.3.1 Details of the procedures used to purify the antibody from culture supernatant fluid, ascitic fluid or antiserum should be documented. Where possible these should be performed with sterile equipment and reagents.

2.3.2. *Contaminants:* the capacity of the purification procedure to remove DNA and RNA should be known. Procedures which are used to destroy or inactivate certain contaminants, such as treatment with deoxyribonucleases, should be efficacious and innocuous.

*2.4 Stability*

Data concerning the binding activity of the antibody and storage conditions (temperature, duration, proteolytic enzyme inhibitors added) should be documented.

*2.5 The final product*

2.5.1 The appearance, colour, form in which presented should be given.

2.5.2 Precise criteria for the purity of the immunoglobulin product should be established. These should include tests with limits for homogeneity (e.g., by a variety of electrophoretic procedures); specification with limits, for specific activity (antibody activity per unit weight protein) and the presence of aggregates.

2.5.3 The final product should be pyrogen-free.

2.5.4 The final product should be negative on culture for bacteria and mycoplasma and should be filtered before administration.

2.5.5 The antibody should be treated to inactivate viruses either in the final product form or at an earlier stage; the method used should be specified and shown to be efficacious and innocuous (e.g., gamma irradiation or heat treatment).

2.5.6 Material from an early batch should be retained as a standard for comparing future batches.

2.5.7 Injected materials should be free of gross aggregates and particles: high speed centrifugation and/or filtration through an appropriate membrane may be necessary.

2.5.8 Details of proposed methods for qualitative or quantitative analysis in body fluids or tissues should be established.

*2.6 Modified antibodies*

For some purposes it may be desirable to modify monoclonal antibodies, e.g., by conjugation with a toxin, radiolabelling, attachment to specific drugs or proteolytic digestion. For such products additional specific control procedures are necessary. These include determination of the molecular weight of the conjugate and evidence of antigen binding activity and of activity, stability and pharmacokinetics of the conjugated drug. Any known incompatibilities with other drugs should be stated.

### 3. Minimum toxicological data for products of hospitals or academic units (for use in a limited number of patients)

### 3.1.1 *Rationale for therapeutic trial*

The rationale for treatment must include:

(a) clear evidence of special or general anti-tumour activity in experimental systems.

*and*

(b) evidence of specific *in vivo* tumour localisation of the antibody in patients with a defined histological tumour type which may reasonably respond to the antibody or drug-antibody conjugate.

*or*

(c) a selective effect on a normal self-renewal system which would reasonably be expected to provide an agent useful against a human tumour recognised as a neoplastic transformation of that self-renewal system or part of it.

### 3.1.2 *Rationale for imaging studies with radiolabelled antibody*

The rationale for imaging studies should include:

1. evidence of localisation of antibody to the appropriate type of tumour cell by immunocytochemistry.

*and/or*

2. evidence of *in vivo* localisation of antibody in a xenograft of the appropriate human tumour in experimental animals.

*and/or*

3. evidence of *in vitro* binding of antibody to a purified tumour associated antigen expressed in the relevant human tumour type.

### *3.2 Toxicity studies*

Appropriate toxicity, histology and haematology will be available following either i.p. or i.v. route depending on the solubility of the agent and the expected mode of administration in the clinic. It is envisaged that studies will be conducted in animals of one sex, usually male.

### 3.2.1 *Unconjugated antibody*

If the purity and specificity (with particular reference to 3.1.1b and c) have been shown to be satisfactory it is expected that the results of single dose toxicity studies should be available. These studies will normally be conducted in mouse (i.e. the species frequently used in antibody production) and one other species, e.g. guinea pig or rabbit. The number of animals required is in the order of 6–10 per dose. The dose given should be 10 times that proposed in man on a *pro rata* basis (e.g. $mg\,kg^{-1}$). In certain circumstances a second dose may be advisable, for example in order to cover the possible need for dose escalation in man. The aim of the study is to identify any untoward or unexpected effect of the antibody. All animals should be sacrificed 14 days after dosing and subjected to gross pathological examination. Histopathology should be carried out on any tissues which are macroscopically abnormal. Haematology will also usually be performed (see Sections 3.2.4 and 3.2.5).

### 3.2.2 *Conjugated antibody*

(a) *Single dose studies* (usually in male animals)
i. *Antibody linked to a known cytotoxic agent* An estimate of the LD10 of the conjugate should be obtained in mice and another species, e.g. guinea-pig or rabbit using 6–10 animals/dose. It is anticipated that the approximate lethality of the cytotoxic agent alone and the antibody alone will be available.

Animals from these studies treated with the LD10 should be observed and weighed for 14 days and then subjected to full histopathological and haematological investigation (see Sections 3.2.4 and 3.2.5).

(a) ii. *Antibody linked to an unknown cytotoxic agent* An estimate of lethality (e.g. LD10) of the cytotoxic agent alone and of the conjugate should be obtained in mice and another species (e.g. rabbit or guinea-pig) using 6–10 animals/dose. Those animals which were treated with the LD10 of the conjugate should be observed and weighed for 14 days and then be subjected to full histopathological and haematological examination (see Sections 3.2.4 and 3.2.5).

(b) *Multiple dose studies* If multiple dose therapy is planned in patients, the estimated LD10 or 10 times the proposed patient dose should be used, for example, in a course of 5 successive daily treatments in mice and one other species (e.g.

rabbit or guinea-pig). Sufficient animals should be used to enable some to be sacrificed at the end of the dosing period and some to be observed and weighed for a subsequent 28 days prior to sacrifice. All animals should be subjected to full histopathological and haematological examination (Sections 3.2.4 and 3.2.5). Should toxicity be evident at this dosage it will be necessary to repeat the experiment using a lower dose in order to ascertain a no or minimal effect level.

3.2.3 *Radiolabelled antibody for diagnostic use*

If the purity and specificity of the antibody have been shown to be satisfactory it is expected that very limited toxicity testing would be conducted. It is suggested that if the radiolabel is replacing a normal atom or molecule, for example tritium replacing hydrogen, then there is no need for studies other than those outlined for the uncojugated antibody (see Section 3.2.1). Where the radiolabel forms an addition product with the antibody it may be advisable to carry out the studies using a cold product prepared in the same way as the radiolabelled product but for example substituting iodine 127 instead of 131. If this is not possible then toxicity studies should be done using the unconjugated antibody only.

3.2.4 *Haematology*

For toxicity studies, a total red cell, white cell and platelet count together with differential assessment of white cell components are to be obtained together with a bone marrow smear.

3.2.5 *Histology*

The following tissues will be examined - bone marrow (squash preparation), kidney, liver, spleen, heart, lung, gut, brain and testis. Any perturbation from normally will be described in detail. Histology of all intercurrent deaths is required.

### 4. Clinical eligibility of patients

In addition to the information outlined below, the following publication is recommended for more detailed considerations:

WHO Handbook for Reporting Results of Cancer Treatment,
WHO Offset Publication No. 48,
World Health Organisation,
Geneva 1979.
Obtainable from HMSO.

4.1 *Physician responsible*

Each patient should be in the care of a cancer physician experienced in management of the relevant tumour type and familiar with investigation of new drugs and management of drug toxicity.

4.2 *Original diagnosis*

Microscopically confirmed diagnosis of cancer.

4.3 *Disease status*

Disease progression despite accepted first line therapy where this exists.

4.4 *Predicted survival*

A predicted survival of not less than 4 months.

4.5 *Treatment status* (for therapeutic studies)

At least a three week interval since the last dose of other potentially myelosuppresive therapy *and* recovery from reversible toxicity.

4.6 *Haematological requirements*

*Minimal haematological requirements:* WBC $>3{,}000\,mm^{-3}$; platelets $>100{,}000\,mm^{-3}$ (excluding patients with acute leukaemia).

4.7 *Biochemical status*

*Minimal biochemical parameters:* normal values for organs related to drug metabolism and excretion or related to major toxic manifestations. Deviations up to 25% above the upper limit of normal liver function will be acceptable however.

4.8 *Consent*

No patient will be entered into the study without his or her consent or that of a parent or guardian for those under 16 years, following a full explanation of the purpose and limited expectations of the study.

4.9 *Contraindication*

Pregnancy, acute intercurrent complications. History of allergy to immunoglobulins of the same species. Positive immediate type hypersensitivity reaction on intradermal testing with antibody. Other concomitant anti-cancer therapy. Performance status: WHO scale 3–4.

### 5. Number of patients to be included

If it is intended to escalate the dose, the following scheme is recommended:

Usually two patients per nontoxic dose level but at least three patients at levels producing limiting toxicity. Seven to ten patients at drug levels with acceptable, reversible toxicity.

### 6. Serial observations

**Appendices 3, 4, 6 and 7** summarise minimum requirements. Additional parameters and/or increased frequency of determinations might be required, depending on animal toxicology and on the needs for the best clinical management of the patient.

### 7. Drug administration

### *7.1 Formulation*

See 2.3–2.5.

### *7.2 Drug administration*

Means to treat anaphylaxis should be available. Route to be stated, e.g. i.v. bolus or infusion over a defined time period.

### *7.3 Initial dose schedule*

The maximum starting dose will be based on 1/10th of the LD10 in mouse given i.v. An arbitrary dose will be needed if the LD10 cannot be attained but 10 times this dose must have been shown to be at or below the LD10 in mice and in a species other than that from which the antibody originates if the antibody is of mouse or rat origin.

### *7.4 Initial drug dose*

Drug doses are given in $\mathrm{mg\,m^{-2}}$ body surface area. Two patients will be entered at the starting dose level (see **Appendix 2**).

### *7.5 Escalation protocol in subsequent patients*

Subsequent patients will be entered at higher dose levels, if no dose-limiting toxicity occurs at the preceding dose level. Normally 3 patients should be entered at each dose level. Consideration may be given to escalating the dose in the same patient or in different patients although these are likely to give different results if patients become immunised to antibody after 1 or more doses.

The dose increments will diminish as the level of expected toxicity is reached and a commonly used escalation protocol is outlined in **Appendix 5**.

### *7.6 Timing of admission of subsequent different patients to treatment*

A time interval of at least 10 days should pass between administration of a drug to one patient and the admission of the next patient at the same dose level.

At least three weeks should pass before entry of further patients into the next higher dose level, in order to take advantage of the experience gained at lower dose levels. The time intervals may be increased as levels of expected drug toxicity are reached.

### *7.7 Dose modification*

Consideration should be given to interrupting medication when there is evidence of hypersensitivity, a major adverse effect of bone marrow except in patients with acute leukaemia or whenever other complications occur.

Medication should also be interrupted or discontinued whenever this is regarded to be in the best interests of the patient, irrespective of toxicity.

### *7.8 Duration of drug administration*

This will have to be established as experience is gained.

### *7.9 Duration of study*

The patients are followed for at least 3 weeks *after the last drug dose*; longer if there is toxicity.

### *7.10 Additional dose schedules*

It may be desirable to explore more than one dose schedule of drug administration (e.g. continuous i.v. infusion or daily 5 day doses).

The starting level for drug dose is 1–2 dose steps below the level of expected toxicity and depends on the experience gained with the initial dose schedule (type of toxicity, reversibility of toxicity, variability of individual tolerance).

### *7.11 Recording of toxicity and response*

**Appendices 3 and 4** give guidelines to be used in addition to history, examination and any other relevant investigation.

### 8. Supportive therapy

Full supportive care and treatment will be given to each patient (antibiotics, transfusions, diet, etc.). Hospitalisation should be recommended for agents with unpredictable toxicity.

*8.1 Radiotherapy*

May be given as a palliative measure. If major areas of haematopoietic tissue need to be irradiated, the patient will be excluded from further study.

*8.2 Additional chemotherapy*

Administration of other antineoplastic agents during the study period disqualifies the patient from the study.

*8.3 Antidotes*

A record of all concurrent non-antitumour drugs used should be provided at the end of the study.

**9. Flow of information and records**

*9.1 Study co-ordinator*

A co-ordinator will be appointed to collate all studies on a single drug within a group.

*9.2 Registration*

Qualifying patients are registered with the study co-ordinator for assignment of drug dose and starting date.

*9.3 Toxicity grading*

See **Appendix 6.**

*9.4 Tumour response*

See **Appendix 7.**

*9.5 Flow sheets*

Flow sheets including tumour measurements will be used to record day to day observations on symptoms, signs and laboratory data.

*9.6 Summary sheet*

See **Appendix 8.**

A summary sheet will be prepared by the study co-ordinator.

*9.7 Sudden toxicity procedure*

The study co-ordinator is called at once in case of pronounced toxicity; he/she will inform the investigators in charge of other patients in the study.

*9.8 Data availability*

The summary form and flow sheets to be made available to the Phase I Clinical Trials Committee at the completion of the study, or at any other time suggested by the Phase I Clinical Trials Committee.

*9.9 Regulatory requirements*

A form for doctors and dentists exemption certificates must be submitted to the DHSS (form MLA 162).

**Appendix 1**

*Tests on cell seed suspension*

Wherever possible tests should be performed on the cell seed suspension to detect the presence of microbial contaminants.

For the isolation of bacteria, fungi and mycoplasma, samples should be inoculated into culture media suitable for the growth of these microorganisms.

For the detection of viruses, samples of disrupted cells, suspended in serum free medium, should be inoculated into 10 suckling mice, of a different strain to those used to prepare the hybridoma cell line where this is of mouse origin, and 5 guinea-pigs. Each mouse should be inoculated i.p. with 0.1 ml of cell suspension. Each guinea-pig should be inoculated i.p. with 2.0 ml. The animals should be observed for at least 4 weeks for evidence of disease or failure to thrive. Such animals should be examined to determine as far as possible the cause of pathology.

If possible, tests for viruses should also be performed by inoculation of cell cultures of human (e.g. MRC-5 cells) and monkey origin (e.g. VERO cells). The cultures should be incubated at 35°C and observed for cytopathogenic changes.

**Appendix 2**

*Useful definitions and conversions*

*Definitions of toxicity* (*1*) HNTD (highest non-toxic dose) – the highest dose at which no haematologic, chemical or morphological drug induced alterations occur.

TDL (toxic dose low = 2 × HNTD) – the lowest dose which produced drug induced pathological alterations in haematological, chemical, clinical or morphologic parameters.

TDH (toxic dose high = 2 × TDL) – the dose which produces drug-induced pathologic alterations in haematologic, chemical, clinical or morphologic parameters.

LD (lethal dose = 2 × TDH) – the lowest dose which produces drug-induced death in any animal during the treatment or observation period.

*In rodents $LD_{10}$ is considered to be the maximum tolerated dose (MTD)*

Conversion of $mg\,kg^{-1}$ to $mg\,m^{-2}$ for different *species*:

$$f \times mg\,kg^{-1} = mg\,m^{-2}$$

| *Species* | (*f*) |
|---|---|
| Mouse | 3.0 |
| Rat | 6.0 |
| Dog | 20.0 |
| Monkey | 12.0 |

*Human*—based on both weight and height. Formula of Dubois and Dubois (3):

$$s = W^{0.425} \times H^{0.725} \times 71.84$$

or $\log S = 0.425 \log W + 0.725 \log H + 1.8564$

$S$ = body surface area in $cm^2$.

$W$ = weight in kg.

$H$ = height in cm.

*Translation of MTD in mice to Phase I starting dose in humans*

It was concluded by Freireich *et al.* (1) and Owens (2) that on a $mg\,m^{-2}$, the MTD was, *on average*, similar in each animal species. It was concluded that: 1/6 $LD_{10}$ in mouse could be used provided this amount is not life threatening or lethal in the dog.

An alternative in the absence of dog toxicity data is to use 1/10th $LD_{10}$ (mouse) that would probably always be tolerated in the dog. The latter is the *recommended* procedure.

**References**

(1) FREIREICH, E.J., GEHAN, E.A., RALL, D.P., SCHMIDT, L.H. & SKIPPER, H.E. (1966). Quantitative comparison of toxicity of anticancer agents in mouse, rat, hamster, dog, monkey, and man. *Cancer Chemotherapy Rep.*, **50,** 219.

(2) OWENS, A.H.J. (1963). Predicting anticancer drug effects in man from laboratory animal studies. *J. Chronic. Dis.*, **15,** 223.

(3) DUBOIS & DUBOIS (1916). *Arch. Intern. Med.*, **17,** 863.

**Appendix 3**

*Clinical, haematological and biochemical data and toxicity related to cytotoxic or other drugs.*

| *Parameter* | *Onset* | *Weekly* | *End of study* |
|---|---|---|---|
| Clinical examination (inc. tumour meas., no) | × | | × |
| Body weight | × | × | × |
| WBC and diff | × | × | × |
| Hb | × | × | × |
| Platelets | × | × | × |
| Bilirubin, Alk, Phos | × | × | × |
| SGOT (SGPT) | × | × | × |
| LDH, Prothrom. time | × | × | × |
| Creatinine | × | × | × |
| Uric acid | × | × | × |
| Urine | × | × | × |
| Chest X-ray | × | every 2–4 weeks | × |
| WHO Performance Status at each visit | × | where indicated | × |
| Blood urea | × | × | × |
| Electrolytes | × | × | × |
| Bone marrow | | where indicated | |

The determination of obligatory and non-obligatory parameters will be made for each agent during the production of each individual protocol.

*Toxicity related to antibody*

| | *Onset* | *Day 1* | *Day 2* | *Weekly* | *End of study (see 7.9)* |
|---|---|---|---|---|---|
| Total IgG | × | | | | × |
| Total IgM | × | | | | × |
| Total IgA | × | | | | × |
| Total complement | × | × | × | × × | × |
| C3 | × | × | × | × × | × |
| C4 | × | × | × | × × | × |
| Rheumatoid factor | × | | | | × |
| Circulating immune complexes | × | × | × | | × |
| Human antibody to administered antibody | × | | | × | × |
| Creatine clearance | × | | | If plasma urea rises | |
| 24 hour urine protein | × | | | If protein on routine testing | |
| Urine red cells | × | | | × | × |
| Urine white cells | × | | | | × |

**Appendix 3—continued**

| | Onset | Day 1 | Day 2 | Weekly | End of study (see 7.9) |
|---|---|---|---|---|---|
| Urine casts | × | | | | × |
| Peak flow | × | × | × | × | × |
| ECG | × | | | after 7 days | × |
| Chest X-ray | × | | | after 7 days | × |
| Clinical history and examination | × | | | × | × |
| *Toxicity related to conjugated substance (131 Iodine in this case)* | | | | | |
| Serum T4 | × | | | Monthly indefinitely | |
| Serum TSH | × | | | Monthly indefinitely | |

**Appendix 4**

*WHO Performance Scale*

| Scale | Performance |
|---|---|
| 0 | Able to carry out all normal activity without restriction. |
| 1 | Restricted in physically strenuous activity but ambulatory and able to carry out light work. |
| 2 | Ambulatory and capable of all self-care but unable to carry out any work; up and about more than 50% of waking hours. |
| 3 | Capable of only limited self-care; confined to bed or chair more than 50% of waking hours. |
| 4 | Completely disabled; cannot carry on any self-care; totally confined to bed or chair. |

**Appendix 5**

*Modified Fibonacci escalation*

| Drug dose | Percentage increase above preceding dose level |
|---|---|
| $n$[a] | — |
| $2n$ | 100 |
| $3.3n$ | 67 |
| $5n$ | 50 |
| $7n$ | 40 |
| $9n$ | 30–35 |
| $12n$ | 30–35 |
| $16n$ | 30–35 |

[a]Starting does $n$ ($mg\,m^{-2}$).

**Appendix 6**

*Definitions of toxicity*

*Toxicity:* Always related to a given dose schedule at a given dose level for a given duration of time.

Grade 0 *Nontoxic dose:* A dose which causes no recognisable effects whatsoever.

Grade 1 *Subtoxic dose:* A dose which causes *consistent* changes of haematological or biochemical or other parameters and might thus herald toxicity at the next higher dose level or with prolonged drug administration.

Grade 2 *Minimal toxic dose:* The smallest dose at which one or more patients show consistent, readily reversible drug toxicity.

Grade 3 *Recommended dose for therapeutic trial:* The dose which causes moderate reversible toxicity in the majority of patients.

Grade 4 *Maximally tolerated dose:* The highest tolerable, safe dosage.

**Appendix 7**

*Tumour response*

Tumour masses are counted and measured at regular intervals. Measurable tumour masses are defined as the product of the longest × the widest perpendicular diameter. The third dimension can be added, where possible. Measurability is arbitrarily defined as reproducibility of simultaneous measurements within 50% by independent observers.

1. Complete tumour regression is defined as complete disappearance of all recognisable tumour masses and/or biochemical changes directly related to the tumour.
2. Complete remission is defined as complete tumour regression (1) *and* complete disappearance of all indirect (host mediated) symptoms, signs, haematological, and biochemical parameters.
3. Partial regression is defined as >50% decrease of one of more tumour lesions in the absence of progression or occurrence of new lesions elsewhere.
4. Improvement applies only to well-outlined palpable lesions, and is defined as >25 to <50% regression in the absence of progression or occurrence of new lesions elsewhere.
5. *Stable disease:* Changes smaller than those outlined under (3), (4) and (6).

6. *Progressive disease:* Occurrence of any new lesion or increase of well-outlined, palpable lesions by 33% or increase of any other measurable lesion by >100% irrespective of regressions elsewhere.

We wish to thank Dr. R.D. Mann, Dr. S. Fawcett and Dr. M.E. Duncan of the Department of Health and Social Security, Market Towers, 1 Nine Elms Lane, London, for their advice in preparation of this document.